# Malaria burden and case management in the Republic of Congo: limited use and application of rapid diagnostic tests results

**DOI:** 10.1186/1471-2458-13-135

**Published:** 2013-02-14

**Authors:** Francine Ntoumi, Jeannhey C Vouvoungui, Rod Ibara, Miguel Landry, Anissa Sidibé

**Affiliations:** 1 Fondation Congolaise pour la Recherche Médicale, Brazzaville, Republic of Congo; 2 Faculty of Health Sciences, University Marien Ngouabi, BP 2672, Brazzaville, Republic of Congo; 3 Institute for Tropical Medicine, University of Tübingen, Tübingen, Germany

**Keywords:** *Plasmodium falciparum*, Malaria, Multiplicity of infection, Rapid diagnostic tests, Microscopy, Republic of Congo

## Abstract

**Background:**

There have been few investigations evaluating the burden of malaria disease at district level in the Republic of Congo since the introduction of artemisinin-based combination therapies (ACTs). The main objective of this study was to document laboratory-confirmed cases of malaria using microscopy and/or rapid diagnostic tests (RDTs) in children and pregnant women attending selected health facilities in Brazzaville and Pointe Noire, the two main cities of the country. Secondly, *P. falciparum* genetic diversity and multiplicity of infection during the malaria transmission season of October 2011 to February 2012 in these areas were described.

**Methods:**

Three and one health facilities were selected in Brazzaville and Pointe-Noire as sentinel sites for malaria surveillance. Children under 15 years of age and pregnant women were enrolled if study criteria were met and lab technicians used RDT and/or microscopy to diagnose malaria. In order to determine the multiplicity of infection, parasite DNA was extracted from RDT cassette and msp2 P.falciparum genotyped.

**Results:**

Malaria prevalence among more than 3,000 children and 700 pregnant women ranged from 8 to 29%, and 8 to 24% respectively depending on health center locality. While health workers did not optimize use of RDTs, microscopy remained a reference diagnostic tool. Quality control of malaria diagnosis at the reference laboratory showed acceptable health centre performances. *P. falciparum* genetic diversity determination using msp2 gene marker ranged from 9 to 20 alleles and remains stable while multiplicity of infection (mean of 1.7clone/infected individual) and parasite densities in clinical isolates were lower than previously reported.

**Conclusions:**

These findings are consistent with a reduction of malaria transmission in the two areas. This study raises the issue of targeted training for health workers and sustained availability of RDTs in order to improve quality of care through optimal use of RDTs.

## Background

Deployment of various control tools has boosted malaria control in sub-Saharan Africa. This includes wide deployment of artemisinin-combination therapies (ACTs), insecticide-treated nets and in some places targeted indoor residual spraying (IRS) of insecticides. Consequently, the burden of malaria has declined in some countries [1], while other countries were unable to provide accurate data on their malaria situation.

The World Health Organization (WHO) recommends parasitological confirmation of malaria using microscopy or RDTs in all malaria suspected patients before starting them on anti-malarial treatment [2]. Treatment based solely on clinical suspicion is warranted when resources for parasitological diagnosis are not available [2,3]. This strategy would ensure the therapeutic longevity of ACTs through protection against development of parasite resistance.

Published data on the burden of malaria from the Republic of Congo since drug policy change in 2002 is limited, making it difficult to evaluate the impact of implemented malaria interventions. Malaria remains the main cause of hospital consultations, accounting for 49 to 51.5% of hospital admissions and 35.4% of children deaths at health facilities in Brazzaville and Pointe-Noire districts [4]. Diagnosis of malaria is through microscopy despite availability of RDTs [5]. At country level, performances of laboratory technicians using microscopy or RDTs are not yet assessed, despite its influence on application of guidelines for malaria treatment and subsequently on the overall impact on malaria control.

Genetic diversity of *Plasmodium falciparum* is an important parameter for malaria surveillance that plays a major role in the natural acquisition of malaria immunity. Exposure to several strains positively influences the development of partial immunity to malaria in people living in malaria endemic areas [6]. Although daily fluctuation of parasite populations does not determine the true individual multiplicity of infection or the genetic diversity of circulating strains [7], the two parameters allow characterization of malaria infection in human populations and improves understanding of acquisition of natural immunity to *P. falciparum*[6].

From 2009, the Central Africa regional network of excellence on HIV/AIDS, tuberculosis and malaria (CANTAM) project engaged itself in passive surveillance of malaria at sentinel sites in Brazzaville and Pointe Noire to provide necessary baseline data. For this purpose, training activities included improving malaria diagnostic skills of laboratory technicians through training together with providing appropriate stocks of RDTs (UNICEF donation) and reagents for microscopy. As most malaria epidemiological studies had been conducted in Southern areas of Brazzaville, the capital of the Republic of Congo [8-10], the main objective of the present study was to document laboratory-confirmed cases of malaria in children and pregnant women attending health facilities in Northern districts of Brazzaville and one main health district Center in Pointe Noire, the second largest city of the Republic. Secondarily, the genetic diversity and multiplicity of *P. falciparum* was determined during the malaria transmission period of October 2011 to February 2012 in these areas. Generated data will form the basis for monitoring and evaluating the impact of introduced malaria control tools.

## Methods

### Study sites

Brazzaville, the capital city of Republic of Congo is served by three health facilities namely: Hospital of Talangai, Centre de Santé Integré (CSI) Fleuve Congo and CSI Intendance in the Northern part of Brazzaville that were selected as sentinel sites for malaria surveillance. CSI Ndaka Sossou health facility in the center of Pointe-Noire, a commercial city bordering the Atlantic Ocean was also selected as a sentinel site. Laboratory technicians were trained for two days, after which they were allowed to participate in the study using microscope or RDTs to diagnose malaria infection. To avoid interference with routine laboratory work, hospital technicians were not involved beyond specimen collection, processing and examination. Patients received antimalarial treatment if result from at least one of the technique was positive.

Malaria transmission in the study areas varies from low, moderate to intense with meso- hyper-to perennial endemicity. There are two rainy seasons each year, from October to December and February to May. In the study by Trape et al. [11], the average entomological inoculation rate (EIR) in Brazzaville was 0.83 infective bite/man/night, due to *Anopheles gambiae*, *A. funestus*, and *A. moucheti.*

#### Ethical clearance

This study was approved by the Institutional Ethics Committee of the Congolese Foundation for Medical Research. Written informed consent was given by participants or by parents or guardians of children who participated in the study.

### Patients

Children under 15 years of age were enrolled into the study if they met the following inclusion criteria: fever (axillary temperature ≥37.5°C at presentation or history of fever within the past 24 hours) and without concomitant infections/diseases, severe malnutrition, or danger signs as defined by WHO [3]. Pregnant women presenting with symptoms of malaria (axillary temperature ≥37.5°C or history of fever and no sign of danger or disease severity) were also enrolled.

### Samples collection

Standard operating procedures for sample collection were provided to the study teams. At inclusion, a rapid diagnostic test (Mal card™) has been performed to diagnose malaria and thick blood smear made to quantify malaria parasites against 200 leucocytes. Parasite density was calculated for each patient assuming an average of 8000 leucocytes per μl of blood. RDT specimens were kept for further parasite DNA extraction.

### Specimen processing

#### Extraction of parasite DNA

Parasite DNA was extracted from RDT cassettes. Briefly, the cassette was broken and the nitrocellulose membrane removed. The visible zones of blood migration on the membrane were cut into small pieces with a scissor and placed in labeled tubes. One ml of lysis solution (sodium phosphate 0.1 g/ml and 0.5% detergent) was added and mixed by vortex for 15 seconds. The samples extracts were incubated for 30 minutes at room temperature (mixed by vortex every 10 minutes for 15 seconds). Samples extracts were then centrifuged at 10 000rpm for 10 minutes. The supernatants were discarded and pellets re-suspended into 50 ul of distilled water. The samples were incubated at 90°C for 10 minutes, mixed by vortex, and 8 000 rpm for 1 minute. The supernatants which include parasite DNA, were removed and conserved at −20°C.

#### Plasmodium falciparum msp2 genotyping

Acknowledging that msp-2 gene is used by many studies as a discriminatory marker for field isolates characterization [12], it is here considered as a single genotyping marker. Genotyping of *falciparum* parasite isolates was performed with nested PCR assays based on the amplification of *msp2* according to Koukouikila et al. [10]. Variable central block 2 was amplified to distinguish allelic families 3D7 and FC27. The amplified PCR products were loaded on to 2% agarose gel (SeaKem, Sigma, St. Louis, MO), subjected to electrophoresis and DNA was visualized under ultraviolet light and photographed to analyze size polymorphism in each allelic family, assuming that one band represented one amplified PCR fragment derived from a single copy of *P. falciparum* MSP2 gene.

#### Distribution of P. falciparum msp2 allele types and multiplicity of infection

The prevalence of the different *msp2* alleles was determined as the presence of PCR products in the total number of *msp2* amplified bands. The multiplicity of infection (MOI) was defined as the mean number of *P. falciparum* genotypes per infected individual. It was calculated as the ratio of the total number of *P. falciparummsp2* genotypes and the number of PCR positive samples.

#### Statistical analysis

Data were double-entered by two clerks, entry discrepancies cleared by supervisor and validated in Epi info version 3.5.1 software by the biostatistician and analyzed using SPSS version 16. Microscopy and PCR were used as the standard references to calculate sensitivity and specificity of different tests at selected health facilities. The variable considered: diagnostic performance were defined using the number of true positive (TP), number of true negative (TN), number of false positive (FP) and number of false positive (FP). The sensitivity of the test was determined as Number of true positives/[Number of true positives + number of false positives] × 100. The specificity of the test was determined as Number of true negatives/ Number of true negatives + number of false positives] ×100.

Positive predictive values (PPVs) and negative predictive values (NPVs) were calculated as described by Iqbal et al. [13].

McNemar-χ^2^ test was used to investigate any significant differences between microscopy and RDT or PCR and RDT. Fisher's Exact test was used to compare malaria prevalence between health centers and also between children and pregnant women. Differences were considered significant if p value<0.05.

The kappa value (κ), representing the proportion of agreement beyond chance, was used to quantify the level of agreement or concordance between both methods; a kappa value of 0.21-0.60 was considered moderate agreement, 0.61-0.80 good agreement and κ ≥ 0.8 was of high concordance [14].

## Results

### Prevalence of malaria infection at the different health facilities

A total of 3,290 children were recruited into the study from four health facilities in Brazzaville and Pointe-Noire (Table 1); out of which, 2,144 were recruited during the transmission season of October to December 2011 Only *P. falciparum* species was detected by microscopy. Four-hundred and four (404) children had malaria infection, representing 12% of consulting cases and reflecting the number of children who received antimalarials. In Brazzaville, CSI Fleuve Congo which is close to Congo river presented the highest malaria prevalence in children: 29% (76/263) compared to 8% (159/2037) and 19% (108/578) at the hospital Talangai and CSI Intendance which are located on a hill. In Pointe-Noire: Out of a total of 412 children, 61 were found microscopy or TDR positive for *Plasmodium falciparum*. The median age of children on consultation at different health care facilities was similar (5 years).

**Table 1:** Malaria positivity by RDTs at various health facilities in Brazzaville and Pointe-Noire during the study period October 2011 to February 2012

**Study size and laboratory tests results**	**Talangaï hospital (%)**	**CSI Fleuve Congo (%)**	**CSI Intendance (%)**	**CSI Ndaka Soussou (%)**	**TOTAL (%)**
**Children< 15 yrs**	2037	263	578	412	3290
**Malaria cases**	159 (8)	76 (29)	108 (19)	61 (15)	404 (12)
**m+/RDT+**	80	27	28	59	194
**m+/RDT-**	79	49	80	2	210
**Mean age years (SD)**	5,78±8,68	6,02±13,62	5,34±3,32	6,20±3,89	-
**Range**	(1–15)	(1–15)	(1–15)	(1–15)	
**Pregnant women**	259	143	335	13	750
**Malaria cases**	22 (8)	27 (19)	79 (24)	3 (23)	131 (17)
**m+/RDT+**	17	6	15	3	41
**m+/RDT-**	5	21	64	0	90
**Mean age years (SD)**	24,36±7,69	26,83±14,16	24,06±4,78	26,39±2,76	-
**Range**	(16–42)	(16–39)	(19–40)	(21–36)	

*m+* microscopy positive; *m*- microscopy negative; *RDT* rapid diagnostic test.

M+/RDT+ both techniques used; m+/RDT- only microscopy used.

*significant difference (p=0.04) between malaria prevalence in children and pregnant women.

Regarding pregnant women, microscopy was mostly used (90/131 cases) and showed malaria prevalence in pregnant women to be similar to that observed in children under 15 years of age in all sites. In details, a total of 750 pregnant women distributed as 737 and 13 were recruited at Brazzaville and Pointe-Noire respectively from October 2011 to February 2012. The mean age of recruited women was 24 years in all selected sites. The prevalence of *P. falciparum* infection in pregnant women ranged from 8 to 24% for all health facilities (Table 1). At the CSI Ndaka Sossou in Pointe –Noire, the prevalence of asymptomatic malaria infections in pregnant women represented 23% (3/13).

### Quality control of laboratory techniques

CSI and CSI Ndaka Sossou preferentially utilized microscopy and RDT for the diagnosis of malaria respectively. Because of this, both district centers were assessed for the microscopy, RDT or PCR at the reference molecular biology laboratory of the Faculty of Health Sciences/University M. Ngouabi in Brazzaville as shown in Table 2. Fifty matched RDT/thick blood smears at CSI Intendance and 27 at Ndaka Sossou were randomly selected and re-read at the reference lab (Table 3). A significant difference in performances between the gold standard PCR and RDT was observed at both centers but no difference when microscopy was considered as gold standard. Kappa values for concordance was moderate (0.43 and 0.35) for microscopy at both health facilities while RDT performance was good (0.88) at CSI Intendance (Table 3).

**Table 2:** **Multiplicity of infection and*msp-2*genetic diversity in*Plasmodium falciparum*isolates from Congolese children attending CSI Intendance (Brazzaville) and Ndaka Sossou (Pointe Noire) health facilities**

**Health facility**	**MOI**	**Nb 3D7 fragments (range size band in bp)**	**Nb FC27 fragments (range size band in bp)**	**Mean parasite density (range)p/μl**
**Brazzaville**	1.6	5 (240–480)	6 (360–460)	87,480 (4000–108800)
**Pointe-Noire**	1.8	13 (300–500)	9 (400–540)	2862 (438–8368)

Nb of fragments = number of PCR fragments using 3D7 or FC27 allele-specific primers.

Mean parasite density = parasite densities of children under five were considered.

**Table 3:** Microscopy and RDT quality control of two health centers in Brazzaville and Pointe-Noire

**Reference lab**							
CSI Intendance		Microscopy			RDT		
		N	P	κ	N	P	κ
	N	41	4	0.43	45	1	0.88
	P	2	3		0	4	
CSI NdakaSossou		Microscopy			RDT		
		N	P	κ	N	P	κ
	N	20	1	0.35	20	1	0.35
	P	4	2		4	2	

The kappa value (κ).

*N* negative, *P* positive.

### P. falciparum multiplicity of infection, genetic diversity and parasite densities in isolates from children in Brazzaville and Pointe-Noire

The multiplicity of infection (MOI) referred as the mean number of ***Plasmodium falciparum*** clones per infected individual, genetic diversity and parasite densities were determined for children consulting in Brazzaville and Pointe-Noire health facilities. MOI was similar (about 1.7) in both health centers. The distribution of 3D7 and FC27 allelic families of msp2 *Plasmodium falciparum* show the presence of 11 and 22 msp2 alleles in clinical isolates from patients from Brazzaville (CSI Intendance) and Pointe Noire (CSI ndaka Sossou) respectively The genetic diversity was much higher in isolates from Pointe-Noire and parasite densities higher in patients from Brazzaville (Table 2).

## Discussion

To bridge knowledge gaps due to limited data from the Republic of Congo on the burden of malaria infection and on utility of available diagnostic tools, the present study documents the burden of uncomplicated malaria and utility of laboratory diagnostics for malaria case management at selected health facilities in Brazzaville and Pointe-Noire, where RDTs and microscopes are already provided. To the best of our knowledge, this is the first study since change of National malaria treatment policy in 2002.

The malaria prevalence of 9% reported in children from two health districts in Northern part of the capital is similar to that reported by Koukouikila *et al.*[10] from Southern part. However malaria prevalence in children living close to the river in Brazzaville and prevalence in Pointe-Noire reached 30% indicating variations in malaria decline and emphasizing need for targeted approaches to control. Despite lack of comparative data for malaria prevalence in pregnant women, the observed 15% prevalence is high and of concern considering that the policy of intermittent preventive treatment with sulfadoxine/pyrimethamine (SP) during pregnancy (SP-IPTp) is implemented throughout the country. Pregnant women were enrolled irrespective of their SP-IPTp status. It is therefore possible that SP-IPTp may not be effective, strengthening need for the re-assessment of this control strategy.

It is interesting noting that at two health centers where quality control for microscopy and interpretation of RDT were done, utility of RDTs as a diagnostic tool was poor particularly in Brazzaville even though laboratory technicians knew. It is unlikely that poor adherence was due to lack of experience. Informally, many technicians were reluctant to use RDTs because their availability was not sustainable and microscope slides were still abundant. Studies conducted in Tanzania [15-17] correctly stressed the fact that the new strategy of malaria confirmation before treatment should be accompanied by systems strengthening to better attain the goal of improving health care. Microscopy reading is time consuming if properly done and it has been reported by many studies that it could be less sensitive than RDT [18]. It is highly possible that Congolese patients have long been treated inappropriately. This study confirms that apart from improving health systems through strengthening logistics, there is also need to invest in manpower development/training and monitoring assessment of processes for implementation.

The genetic diversity among *Plasmodium falciparum* field isolates in Northern Brazzaville and in Pointe-Noire reveals limited and high genetic diversity but not surprising considering that CSI Intendance is located on a hill. In comparison the genetic diversity in Southern part of Brazzaville where malaria transmission is intense, the MOI and genetic diversity [10] was higher. The genetic diversity in Pointe Noire remained high despite known introduced intervention tools suggesting lack of impact. However, this might be explained by differences in laboratory techniques that were used in the two studies to determine genetic diversity [19]. Unfortunately, entomological studies have not been carried out in these areas to determine the current level/intensity of malaria transmission. In earlier studies conducted in Southern area of Brazzaville, the MOI was 2.2 in clinical isolates [9] and reduced to 1.6 after introduction of ACTs. Similar MOI is now reported from the Northern part of Brazzaville and from Pointe-noire. A similar trend in reduction of parasite densities is observed from Southern part of Brazzaville when current findings are compared to those published earlier [19-21]. This may reflect an impact of the ACTs and high use of insecticide-treated nets within the country. However, the genetic diversity remains unchanged suggesting that introduced interventions do not eliminate specific allelic forms.

We recommend that laboratory technicians should be trained on the importance and advantages of using RDTs compared to microscopy such as simplicity, patient time saved and its modest reliability. Indeed, malaria is over-diagnosed and over-treated in many African countries through clinical diagnosis only, while other clinicians do not honor laboratory results [22-24]. While it is clear that behavior change is a lengthy process, the notion that health workers were rather skeptical about a steady supply of RDTs by the state sounds lame.

## Conclusion

The study has shown the need to provide a targeted training to health workers to enhance acceptability of RDTs and improve quality of care. It also revealed that MOI and parasite densities in clinical Congolese isolates from the two main cities are low and the *P.falciparum* genetic diversity remains stable.

## Abbreviations

RDT: Rapid diagnostic test; IRS: Indoor residual spray; PCR: Polymerase chain reaction; SP: Sulfadoxine pyrimethamine; IPTp: Intermittent preventive treatment during pregnancy; MOI: Multiplicity of infection.

## Competing interest

The authors declare no competing interest.

## Authors’ contributions

FN, JCV and AS contributed to the study design, data analysis and drafting of the manuscript. ML and RI performed the lab work. JCV performed the statistical analysis. FN ensured the supervision of the all work. All authors read and approved the final manuscript.

## Pre-publication history

The pre-publication history for this paper can be accessed here:

http://www.biomedcentral.com/1471-2458/13/135/prepub

## References

[B1] World Health Organization malaria report 2011 Geneva: WHO

[B2] WHO Guidelines for the treatment of malaria 2010 2 Geneva: WHO 25473692

[B3] World Health Organization WHO/HTM/MAL/2006.1111 WHO The role of laboratory diagnosis to support malaria disease management: focus on the use of rapid diagnostic tests in areas of high transmission. Report of WHO technical consultation 2006 Geneva: WHO

[B4] Ministère de la Santé Publique03/10/2010

[B5] WHO Malaria rapid diagnostic tests performance. Results of WHO products testing of malaria RDTs round 2 2009

[B6] Conway DJ Cavanagh DR Tanabe K A principal target of human immunity to malaria identified by molecular population genetic and immunological analyses Nature Med 2000 6 6 689 92 10.1038/76272 10835687

[B7] Färnert A Lebbad M Faraja L Extensive dynamics of Plasmodium falciparum densities, stages and genotyping profiles Malaria J 2008 7 241 10.1186/1475-2875-7-241 PMC2605469 19025582

[B8] Ndounga M Mayengue PI Tahar R Efficacy of sulfadoxine-pyrimethamine, amodiaquine, and sulfadoxine-pyrimethamine-amodiaquine combination for the treatment of uncomplicated falciparum malaria in the urban and suburban areas of Brazzaville (Congo) Acta Tropica 2007 103 3 163 71 10.1016/j.actatropica.2007.06.002 17645863

[B9] Mayengue PI Ndounga M Malonga FV Genetic polymorphism of merozoite surface protein-1 and merozoite surface protein-2 in Plasmodium falciparum isolates from Brazzaville, Republic of Congo Malaria J 2011 10 1 276 10.1186/1475-2875-10-276 PMC3195763 21936949

[B10] Koukouikila-Koussounda F Malonga V Mayengue PI Genetic polymorphism of merozoite surface protein 2 and prevalence of K76T pfcrt mutation in Plasmodium falciparum field isolates from Congolese children with asymptomaticinfections Malaria J 2012 11 1 105 10.1186/1475-2875-11-105 PMC3349535 22463364

[B11] Trape JF Zoulani A Malaria and urbanization in Central Africa: the example of Brazzaville. Part II: Results of entomological surveys and epidemiological analysis Trans Roy Soc Trop Med and Hyg 1987 81 Suppl 2 10 18 2901796 10.1016/0035-9203(87)90472-x

[B12] Ntoumi F Ngoundou-Landji J Lekoulou F Site-based study on polymorphism of P. falciparum MSP-1 and MSP-2 genes in isolates from two villages in Central Africa Parassitologia 2000 42 197 203 11686079

[B13] Iqbal S Iqbal R Khan MM Comparison of two conventional techniques used for the diagnosis of tuberculosis cases Int J Ag and Biol 2003 5 4 545 547

[B14] McGinn T Wyer PC Newman TB Tips for learners of evidence –based medicine: 3. Measures of observer variability (Kappa statistic) Canadian Med As J 2004 171 11 1369 1373 10.1503/cmaj.1031981 PMC527344 15557592

[B15] Masanja IM de Bethune X Jacobs J Implementing ideal health policy in a fragile health system: the example of expanding the use of malaria rapid diagnostic tests in mainland Tanzania Malaria J 2011 28 10,322 10.1186/1475-2875-10-322 PMC3212840 22035466

[B16] Masanja IM Selemani M Amuri B Increased use of malaria rapid diagnostic tests improves targeting of anti-malarial treatment in rural Tanzania: implications for nationwide rollout of malaria rapid diagnostic tests Malaria J 2012 11 1 221 10.1186/1475-2875-11-221 PMC3471012 22747655

[B17] Chinkhumba J Skarbinski J Chilima B Comparative field performance and adherence to test results of four malaria rapid diagnostic tests among febrile patients more than five years of age in Blantyre, Malawi Malaria J 2010 9 209 10.1186/1475-2875-9-209 PMC2916916 20646312

[B18] Kattenberg JH Tahita CM Versteeg IAJ Evaluation of antigen detection tests, microscopy, and polymerase chain reaction for diagnosis of malaria in peripheral blood in asymptomatic pregnant women in Nanoro, Burkina Faso Am J Trop Med and Hyg 2012 87 2 251 256 10.4269/ajtmh.2012.12-0125 22859362 PMC3414559

[B19] Durand P Michalakis Y Cestier S Significant linkage disequilibrium and high genetic diversity in a population of Plasmodium falciparum from an area (Republic of the Congo) highly endemic for malaria Am J Trop Med Hyg 2003 68 3 345 9 12685643

[B20] Ndounga M Tahar R Basco LK Therapeutic efficacy of sulfadoxine–pyrimethamine and the prevalence of molecular markers of resistance in under 5-yearolds in Brazzaville, Congo Trop Med and Int Health 2007 12 10 1164 1171 10.1111/j.1365-3156.2007.01904.x 17956498

[B21] Mayengue PI Ndounga M Matondo Maya D Ntsonde T Ntoumi F In vivo chloroquine resistance and prevalence of the pfcrt codon 76 mutation in Plasmodium falciparum isolates from the Republic of Congo Acta Tropica 2005 95 219 225 10.1016/j.actatropica.2005.06.001 16002038

[B22] Mtove G Nadjm B Amos B Use of an HRP2-based rapid diagnostic test to guide treatment of children admitted to hospital in a malaria-endemic area of north-east Tanzania Trop Med and Int Health 2011 16 5 545 50 10.1111/j.1365-3156.2011.02737.x 21320243 PMC3469736

[B23] Mtove G Hendriksen ICE Amos B Treatment guided by rapid diagnostic tests for malaria in Tanzanian children: safety and alternative bacterial diagnoses Malaria J 2011 10 290 10.1186/1475-2875-10-290 PMC3210100 21978238

[B24] Hendriksen ICE Mtove G Pedro AJ Evaluation of a PfHRP2 and a pLDH-based Rapid Diagnostic Test for the Diagnosis of Severe Malaria in 2 Populations of African Children Clin Inf Dis 2011 52 9 1100 7 10.1093/cid/cir143 PMC3070869 21467015

